# SPICE-19: a 3-Month Prospective Cohort Study of 640 Medical Students and Foundation Doctors

**DOI:** 10.1007/s40670-021-01349-0

**Published:** 2021-07-21

**Authors:** Soham Bandyopadhyay, Ioannis Georgiou, Emily Bligh, Conor Coyle, Rohan Pancharatnam, Kate E. A. Saunders, The NANSIG Collaborative, Marta de Andres Crespo, Ashok Handa, Conor S. Gillespie, Bibire Baykeens, Mohammed Talha Bashir, Maria Georgiou, Shumail Mahmood, Anna Casey, Rosalind Di Traglia, Alex Fung, Jack Wellington, Adam Hounat, Jay J. Park, Joshua Erhabor, Mohammad H. Ashraf, Hanya Ghazi, Lucas M. Hernandez, Zeluleko Sibanda, Makinah Haq, Salma Mahmood, Abbey Boyle, Carlos M. Curtis-Lopez, Harry James Carr, Lorcán McMullan, Michael McLarnon, Armin Nazari, Emma Jane Norton, Guan Hui Tricia Lim, Oliver Rushworth

**Affiliations:** 1 Nuffield Department of Surgical Sciences, Oxford University Global Surgery Group, University of Oxford, Oxford, UK; 2 The School of Medicine, University of Aberdeen, Aberdeen, UK; 3 Faculty of Medicine, Dentistry & Health, University of Sheffield Medical School, Sheffield, UK; 4 St George’s, University of London, Cranmer Terrace, London, UK; 5 The University of Buckingham Medical School, The University of Buckingham, Milton Keynes, UK; 6 Department of Psychiatry, Warneford Hospital, University of Oxford, Warneford Lane, Oxford, UK; 7 http://www.nansig.org

**Keywords:** Coronavirus, COVID-19, Medical education, Mental health, Medical students, Prospective study

## Abstract

**Introduction:**

There is paucity of data around the support that medical students have been provided with, need to be provided with, and would like to be provided with during the COVID-19 pandemic. This study sought to explore the effects of the COVID-19 pandemic on medical students and establish the support they require.

**Methods:**

A prospective, observational, multicentre study was conducted in 2020. All medical students and interim foundation year 1 doctors were eligible to participate.

**Results:**

Six hundred forty individuals participated from 32 medical schools. Participants reported a drop in their mood following the onset of the pandemic (*p* < 0.001). This drop in mood was evident in both May and August. Participants did have an improved mood in August compared to May (*p* < 0.001). There was a significant decrease in pandemic disease-anxiety (13.8/20 to 12.4/20, *p* < 0.001) and consequence-anxiety (6.3/10 to 6.0/10, *p* < 0.001) between May and August. Nineteen percent of participants (*n* = 111/596, 19%) had not received the support they needed from their university by August. The most common area of support that our participants needed and had not received from their medical schools by August was support with course material (*n* = 58/111, 52%). ‘Clinical knowledge’ was thought to have been affected by the greatest number of participants in both May and August.

**Conclusion:**

Medical students’ mental well-being has been adversely affected during the COVID-19 pandemic. Our findings have actionable implications that can better protect medical students as they acclimatise to a working environment that has been radically changed by COVID-19.

**Supplementary Information:**

The online version contains supplementary material available at (10.1007/s40670-021-01349-0).

## Introduction

The severe acute respiratory syndrome coronavirus 2 (SARS-CoV-2) is responsible for the global pandemic known commonly by the disease name COVID-19 [1, 2]. This has led to the greatest level of collective uncertainty in living memory [3] and responses to limit the spread of the virus have led to substantial lifestyle adaptations [4]. It is clear that for both the immediate and the long-term future, support is needed for the population at large [5–8]. Support is a broad concept encapsulating assistance, aid, or empowerment of individuals. Tangible academic, financial, and emotional support is absolutely essential for individuals’ well-being [9]. However, the nature of this support is likely to vary at an individual and community level. At present, there is a paucity of data characterising the support that individuals have been provided with, need to be provided with, and would like to be provided with. This is compounded by the fact that some types of support may be less likely to be provided to certain population sub-groups [10, 11], and similarly some areas of support may be less likely to be requested for by certain population sub-groups [12].

One population sub-group of interest that has been particularly in need of support during the COVID-19 pandemic are medical students. Medical students at all stages of training have been significantly impacted by the COVID-19 pandemic [13]. Students have faced substantial changes and challenges to their education including the suspension of clinical placements, changes to assessments, and closure of campuses [14]. Such measures have removed students from established structures and limited the ability of students to access sources of support over a period of potential need [15, 16]. A proportion of medical students in 2020 graduated early and took on the novel job role as an interim foundation year 1 (FiY1) doctor to help alleviate NHS pressures through the pandemic [17]. Those who were granted early provisional registration bridged the time gap in employment between starting as a Foundation Year 1 Doctor (FY1 or US equivalent to PGY1) in August 2020. However, concerns were raised that FiY1 doctors and volunteering medical students who took to take on front-line roles may not have had sufficient training on infection prevention and control (IPC) [18, 19], and sufficient information on [20]or access to adequate personal protective equipment (PPE) [21]. The latter issue is of particular concern given that a lack of appropriate PPE has been identified as a risk factor for COVID-19-related death in healthcare workers [22, 23].

Medical students are already known to suffer with increased rates of anxiety, feelings of pressure and burnout compared to the general population [24, 25]. Given the added stressors associated with the COVID-19 pandemic, there is a risk that increasing numbers of medical students will develop anxiety, depression or self-injurious behaviours [26]. Therefore, it is imperative that support needed by medical students is identified and provided in order to minimise the risk to their mental and physical health. A lack of support for current medical students could have wider repercussions. For example, if medical students decide not to progress with their medical degree due to the feelings of being unsupported, this will pose a significant risk to future medical workforce planning and thereby healthcare service delivery.

The Social and Psychological Impact of COVID-19 on medical students: a national survey Evaluation (SPICE-19) was a prospective study with the following research question: what support did medical students and FiY1 doctors across the United Kingdom (UK) receive and seek during the COVID-19 pandemic. This study primarily aimed to explore whether participants’ mood was affected during the COVID-19 pandemic, how they were affected, and identify relevant contributory factors. The secondary aims of the study were to categorise the types and areas of support (e.g. academic, financial and emotional) that medical schools provided, as well as the support sought by medical students and FiY1 doctors. This “magnified” snapshot of elements impacting student doctor wellness and readiness for practice offers insights into what schools and early career support structures may evolve into to improve the long known gaps in these areas. It is hoped that our study results ensure the development of long-term policy changes that promote the psychological and social well-being of students and healthcare workers.

## Methods

### Study Design

The SPICE-19 study was a national, multicentre, prospective, observational cohort study, which has been conducted in line with the pre-specified protocol. An initial questionnaire (Appendix S1) was disseminated to medical students and FiY1 doctors between May 4, 2020, and May 31, 2020. One regional lead from each medical school was responsible for ensuring that the questionnaire was disseminated via email and social media at least once per week during the time it was open. Regional leads were required to provide evidence that they had circulated the questionnaire weekly to qualify for collaborative authorship. Dissemination was also performed through collaborative networks: the International Student Surgical Network (InciSioN) UK [27], and the Neurology And NeuroSurgery Interest Group (NANSIG) [28]. On completion of the initial questionnaire, a report was created about the support that medical students and FiY1 doctors needed [29]and circulated to medical schools. The purpose of the real-time feedback was to optimise the support provided at a time where it could have most impact. Participants who consented for further contact were directly emailed a link to complete the follow-up questionnaire (Appendix S2) between August 4, 2020, and August 31, 2020, to assess if their support needs had been met or had changed. Reminders were publicised on social media by regional leads and collaborative networks. The study received ethical approval by the University of Oxford Medical Sciences Inter-Divisional Research Ethics Committee (Ethics Approval Reference: R69297/RE001) on the of April 16, 2020. The Strengthening the Reporting of Observational Studies in Epidemiology (STROBE) statement was used in the preparation of this section of the manuscript [30].

The study was co-developed with medical students and FiY1 doctors. It underwent a series of iterations whereby amendments were made, and feedback sought. An initial qualitative data gathering exercise was conducted of 170 medical students and FiY1 doctors that had graduated from the University of Oxford [31]. Based on the concerns and thoughts raised by these individuals, the questions for both the initial and follow-up study were created. However, we were not confident that views of medical students and FiY1 doctors in one medical school in the country would be representative of the population in question. As such, the questionnaire was sent to medical students across the country. At least one individual at all 34 medical schools in the UK provided feedback on the design, content and usability of the questionnaire. Checking the responses from different individuals at different medical schools was used to establish inter-rater reliability. In response to the feedback, the questionnaire was updated to improve clarity and objectivity. The updated questionnaire was resent twice at two different time points to a sample of medical students to ensure intra-rater reliability. More than 90% of the responses being the same was taken to be a sign of reliability.

### Eligible Participants

Any individual enrolled in a medical school in the UK recognised by the GMC and listed by the Medical School Council (MSC) at the start of the 2019/2020 academic year (Appendix S3) was eligible to participate. A majority of these students are White and Female [32]. The exclusion criteria included individuals below the age of 18, and individuals unwilling or unable to give informed consent. As a result of a similar study being conducted locally at one eligible medical school, responses from their enrolled medical students and recently graduated FiY1 doctors were excluded from the study.

### Data Collection

Data points collected were participant demographics, the well-being of participants, and the factors perceived to affect their well-being. The well-being of participants was assessed using two validated scales: the pandemic anxiety scale (PAS) [33]and the Warwick-Edinburgh Mental Wellbeing Scale (WEMWBS) [34], as well as a third, non-validated mood scale that ranged from 0 (lowest mood) to 100 (highest mood). Mood is an affective state, and a component of subjective well-being [35]. The construct of mood has been recognised as a spectrum of activation where individuals have moods that can range anywhere from high (100forthepurposesofthisstudy) to low (0 for the purposes of this study) [35]. Given evidence that individuals in lower mood states than their baseline can accurately recall the past [36], and our hypothesis that individuals surveyed would have a lower mood than their baseline state, individuals answering the original questionnaire in May were asked to recall their mood prior to the pandemic. Their answer to this question was not available when they answered the question about their mood in August. In the initial questionnaire, Likert item 7 of the original 9-item PAS (a question related to missing school) was excluded as it related to a paediatric population. In between the initial survey and follow-up survey, the PAS was validated. Following the validation of the PAS, a final 7-item PAS was created that used a modified wording of the original Likert item 7. The final 7-item PAS was divided into a 4-item disease anxiety sub-scale and a 3-item consequence anxiety sub-scale. All 4 Likert items of the disease anxiety sub-scale were present in the initial and follow-up questionnaires. Only 2 out of the 3 Likert items of the consequence anxiety sub-scale were present in the initial and follow-up questionnaires due to exclusion of the original Likert item 7. For each Likert item on the PAS, strongly disagree was given 1 point and strongly agree was given 5 points (with 2, 3 and 4 points given to the variables in the middle). The WEMWBS was only utilised in the follow-up questionnaire. For the initial questionnaire, priority was given to those areas identified as most relevant by students. Data capture was undertaken using a self-reported online survey tool on the Qualtrics™ platform and only authorised members of the research team had access to the research data.

### Data Analysis

The data was reported using descriptive statistics. Where participants indicated that they would prefer not to answer, they have been removed from the analyses of that section. *p* Values were calculated using the two-tailed paired Student’s *t*-test and the Wilcoxon signed rank test for parametric and non-parametric data, respectively. The relationship between WEMWBS scores and PAS scores or mood scores was identified using a multiple linear regression model. The McNemar’s test was used to determine if there were a significant difference between self-reported sufficiency of PPE information and IPC training between May and August. The assumptions of the statistical methods used were met. Graphpad Prism 5™ statistical software was used.

## Results

### Demographics

Two thousand seventy-five individuals from all 34 eligible UK medical schools were asked to participate in this prospective study. Six hundred forty (30.8%) individuals from 32 medical schools agreed to participate. This represented 94% (*n* = 32/34) of the medical schools in the UK recognised by the GMC and listed by the MSC at the start of the 2019/2020 academic year. Five hundred ninety-three medical students (*n* = 593/640, 93%) and 47 FiY1 doctors (*n* = 47/640, 7%) were recruited in May. Participants were equally spread across all stages of training (Table 1). The majority of our participants were female (*n* = 500/640, 78%) and white (*n* = 468/640, 73%).

**Table 1:** Demographics of participants as of May 2020

Demographics	Number
Stage of training	Medical Students: Year 1 (excluding intercalated year): 80 Year 2 (excluding intercalated year): 117 Year 3 (excluding intercalated year): 122 Year 4 (excluding intercalated year): 159 Year 5 (excluding intercalated year): 114 Prefer not to answer: 1 FiY1 doctors: 47
Age	Median Age: 22 (range: 18 – 37)
Gender	Male: 130 Female: 500 Non-binary: 4 Prefer not to answer: 6
Ethnicity	White: 468 • British: 407 • Irish: 10 • Other White: 51 Mixed/multiple ethnic groups: 28 • White and Black Caribbean: 3 • White and Asian: 11 • White and Black African: 1 • Other Mixed: 13 Asian/Asian British: 102 • Indian: 50 • Pakistan: 12 • Bangladeshi: 3 • Chinese: 17 • Other Asian: 20 Black/African/Caribbean/Black British: 23 • African: 14 • Caribbean:4 • Other ethnic group: 11 Prefer not to answer: 13

### Mood of Participants

**Fig. 1:**
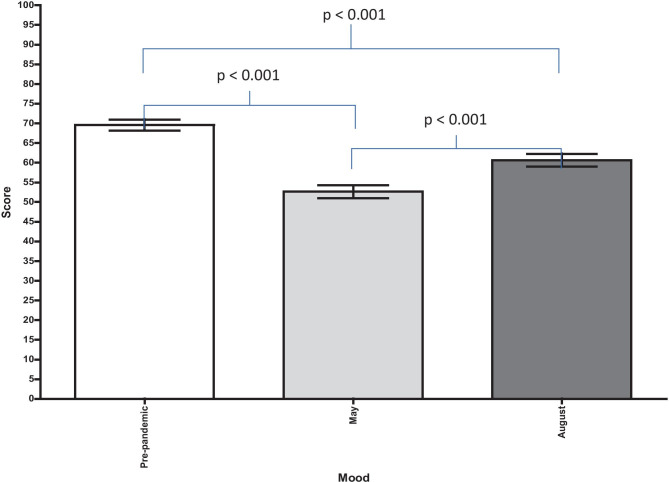
Participants’ mean reported mood at three timepoints. Pre-pandemic, 69.6 [95% CI 68.2, 71.0]. May, 52.7 [95% CI 51.0, 54.3]. August, 60.7 [95% CI 59.0, 62.3]

Overall, participants reported a drop in their mood following the onset of the pandemic (*p* < 0.001). This drop in mood was evident in both May and August (Fig. 1). Participants did report an improved mood in August compared to May (*p* < 0.001). Both medical students and doctors were similarly affected. There was a significant association and positive correlation between participants’ reported mood in August and their WEMWBS scores (*p* < 0.001). Five hundred seventy-nine participants (*n* = 579/640, 90%) described the factors that had negatively and positively influenced their mood in both May and August (Table 2). Compared to May, there was a general increase in the number of participants citing each factor surveyed as a positive influence on mood and a general decrease in the number of participants citing each factor surveyed as a negative influence on mood in August. However, there was an increase in the number of participants citing the strain of wearing protective equipment (*p* < 0.001) and the high demands in the work setting as having a negative influence on their mood (*p* = 0.035). The demographic group that saw the greatest proportional increase in individuals citing the above two factors as having a negative influence on their mood were medical students that were in their final year in May, where there was a threefold increase.

**Table 2:** Factors affecting mood in participants

Factors that had a positive influence	% Study participants who report a positive influence on mood (*n* = /579) in May	% Study participants who report a positive influence on mood (*n* = /579) in August
More time at home/with family	68 (393)	69 (400)
Reduced responsibilities/more free time	59 (342)	54 (314)
Participant and family members remained unaffected from COVID-19	56 (323)	60 (346)
Time away from work/university	46 (269)	56 (323)
Cancelled/open book exams	43 (247)	38 (218)
Opportunities for paid work	24 (138)	27 (159)
Improvements in physical health	20 (113)	22 (126)
Online learning	19 (109)	24 (137)
Improved financial status	14 (83)	18 (104)
Volunteering opportunities	13 (73)	11 (63)
Increase in research opportunities	4 (26)	5 (27)
Relaxation of measures imposed to control the spread of COVID-19		51(298)
Reduction in COVID-19 cases in the UK		43 (250)
Return to face-to-face teaching		11 (65)
More medical school teaching		5 (27)
Other factors	4 (23)	2 (13)
Factors that had a negative influence on mood	% Study participants who report a negative influence on mood (*n* = /579) in May	% study participants who report a negative influence on mood (*n* = /579) in August
Social distancing	81 (467)	67 (388)
Reports on social media and news outlets	73 (424)	73 (424)
Self-isolation	59 (344)	52 (303)
Holiday cancelled	53 (307)	46 (265)
Social exclusion	41 (235)	33 (191)
Financial worries	29 (165)	31 (177)
Career uncertainty	27 (155)	17 (99)
Relatives or friends getting infected	20 (117)	13 (75)
Elective cancelled	18 (105)	18 (105)
Deterioration of physical health	13 (76)	12 (68)
Reduction in research opportunities	12 (70)	12 (72)
Strain of wearing protective equipment	9 (51)	17 (97)
High demands in the work setting	9 (50)	12 (72)
Recent bereavement of someone you know from COVID-19	7 (40)	7 (39)
Stigmatisation	7 (40)	7 (38)
Getting infected	5 (31)	6 (32)
Uncertainty related to medical education		67 (388)
Changes to medical education		66 (384)
Uncertainty related to examinations		60 (345)
Other factors	17 (99)	8 (46)

### Pandemic-Anxiety of Participants

Of the 640 participants, 617 (n = 617/640, 96%) provided a PAS score in both May and August. In May, the mean PAS score was 26.8/40 [95% CI 26.5, 27.1]. In August, the mean PAS score was 21.8/35 [95% CI 21.4, 22.1]. Among the 632 individuals that completed the PAS in August, there was a significant association and negative correlation between their PAS scores and WEMWBS scores (*p* < 0.001).

### Disease-Anxiety of Participants

**Fig. 2:**
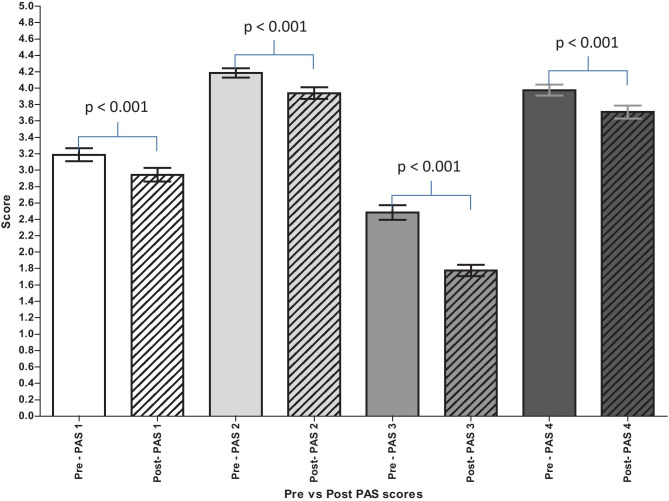
Mean scores (with 95% confidence intervals) for Likert items that make up the disease-anxiety segment of the PAS. Pre refers to scores in May. Post refers to scores in August. PAS 1: I am worried that I will catch COVID-19. PAS 2: I am worried that friends and family will catch COVID-19. PAS 3: I am afraid to leave the house right now. PAS 4: I am worried I might transmit the infection to someone else

The average score of the disease-anxiety segment of the PAS decreased from 13.8/20 [95% CI 13.6, 14.0] in May to 12.4/20 [95% CI 12.1, 12.6] in August (*p* < 0.001). This decrease was reflected in the mean scores of all the Likert items that made up the disease-anxiety segment of the PAS (Fig. 2 and Table 3). For all the aforementioned Likert items, both medical students and FiY1 doctors reported a significant decrease in their disease-anxiety (*p* < 0.05). Medical students that were in their final year in May did not have a significantly decreased score for the Likert item: ‘I am worried that I will catch COVID-19’ (*p* = 0.327; 3.2/5 to 3.0/5). Participants that identified as ‘Asian/Asian British’ did not have a significantly decreased score for the Likert items: ‘I am worried that I will catch COVID-19’ (*p* = 0.386; 3.3/5 to 3.2/5) and ‘I am worried I might transmit the infection to someone else’ (*p* = 0.115; 3.9/5 to 3.7/5). Participants that identified as ‘Black/African/Caribbean/Black British’ did not have a significantly decreased score for the Likert items: ‘I am worried that I will catch COVID-19’ (*p* = 0.690; 2.9/5 to 3.1/5), ‘I am worried that friends and family will catch COVID-19’ (*p* = 1.000; 3.9/5 to 3.9/5) and ‘I am afraid to leave the house right now’ (*p* = 0.158; 2.6/5 to 2.3/5).

**Table 3:** Breakdown of data for Likert items

Likert item	% Study total who disagree (*n* = /617)		% Study total who agree (*n* = /617)		Mean score out of 5 [95% CI]	
	May	August	May	August	May	August
I am worried that I will catch COVID-19*	34 (207)	38 (236)	51 (316)	36 (221)	3.2 [3.1, 3.3]	2.9 [2.9, 3.0]
I am worried that friends and family will catch COVID-19*	5 (28)	9 (58)	91 (563)	78 (484)	4.2 [4.1, 4.2]	3.9 [3.9, 4.0]
I am afraid to leave the house right now*	63 (391)	85 (522)	29 (178)	7 (41)	2.5 [2.4, 2.6]	1.8 [1.7, 1.8]
I am worried I might transmit the infection to someone else*	9 (56)	15 (91)	85 (527)	71 (438)	4.0 [3.9, 4.0]	3.7 [3.6, 3.8]
I am worried about the amount of money we have coming in**	55 (340)	55 (338)	36 (222)	28 (175)	2.7 [2.6, 2.8]	2.6 [2.5, 2.7]
I am worried about the long-term impact this will have on my job prospects and the economy**	22 (133)	26 (161)	69 (426)	56 (346)	3.6 [3.5, 3.7]	3.4 [3.3, 3.5]

^*^Likert items that make up the disease-anxiety segment of the PAS

^**^Likert items that make up the consequence-anxiety segment of the PAS

### Consequence-Anxiety of Participants

**Fig. 3:**
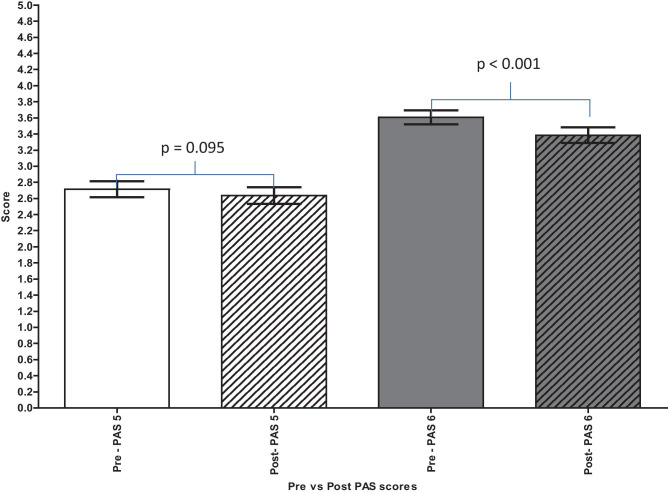
Mean scores (with 95% confidence intervals) for Likert items that make up the consequence-anxiety segment of the PAS. Pre refers to scores in May. Post refers to scores in August. PAS 5: I am worried about the amount of money we have coming in. Pas 6: I am worried about the long-term impact this will have on my job prospects and the economy

The average score of the consequence-anxiety segment of the PAS decreased from 6.3/10 [95% CI 6.2, 6.5] in May to 6.0/10 [95% CI: 5.9, 6.2] in August (*p* < 0.001). This decrease was reflected in one of the two Likert items that made up the consequence-anxiety segment of the PAS (Table 3 and Fig. 3): ‘I am worried about the long-term impact this will have on my job prospects and the economy’ (*p* < 0.001). The score for this Likert item did not significantly decrease in several demographic sub-groups: FiY1 doctors (*p* = 0.465; 3.3/5 to 3.2/5), participants that identified as male (*p* = 0.535; 3.3/5 to 3.2/5) and participants that identified as ‘Black/African/Caribbean/Black British’ (*p* = 1.000; 3.4/5 to 3.4/5). Females were the only demographic sub-group to have a significantly decreased score for the Likert item: ‘I am worried about the amount of money we have coming in’ (*p* = 0.004; 2.8/5 to 2.7/5).

### Support Provided to Participants by Medical Schools

Of the 640 participants, 596 participants (*n* = 596/640, 93%) reported on the support that their university had provided during both stages of this study. The majority of participants (*n* = 504/596, 85%) reported that their university had provided some form of support by May. The three most common forms of support that individuals reported had been provided were written support (*n* = 395/596, 66%), support with course material (*n* = 294/596, 49%), and online material or videos for self-support (*n* = 285/596, 48%). One hundred and ten participants (*n* = 110/504, 22%) stated that the support provided had not been useful. Approximately half of the participants (*n* = 295/596, 49%) wanted more support from their university. The five most common areas of further support sought by our participants from their medical schools in May were support with exam preparation (*n* = 172/295, 58%), support with course material (*n* = 166/295, 56%), financial guidance (*n* = 100/295, 34%), online face-to-face support (*n* = 99/295, 34%) and online material or videos for self-support (*n* = 86/295, 29%). Table 4 details the support that medical students—by year group—wanted from their medical school in May.

**Table 4:** Support sought by medical students by year in May and August. These values do not total 100% as some participants sought multiple forms of support and other participants sought no support

	Stage of training									
	% of study total who sought support in May					% of study total who sought support in August				
	Year 1 (*n* = /75)	Year 2 (*n* = /110)	Year 3 (*n* = /113)	Year 4 (*n* = /149)	Year 5 (*n* = /105)	Year 1 (*n* = /75)	Year 2 (*n* = /110)	Year 3 (*n* = /113)	Year 4 (*n* = /149)	Year 5 (*n* = /105)
Online material/videos for self-support	12 (9)	15 (16)	16 (18)	15 (22)	14 (15)	4 (3)	6 (7)	10 (11)	12 (18)	6 (6)
Online face-to-face support	11 (8)	17 (19)	15 (17)	17 (26)	24 (25)	3 (2)	2 (2)	8 (9)	5 (7)	4 (4)
Letters, emails, or any other form of written support	1 (1)	10 (11)	9 (10)	10 (15)	12 (13)	1 (1)	4 (4)	4 (4)	9 (13)	8 (8)
Financial guidance	9 (7)	16 (18)	14 (16)	21 (31)	21 (22)	3 (2)	5 (6)	3 (3)	5 (8)	4 (4)
Information on COVID-19 symptoms	4 (3)	3 (3)	0 (0)	1 (2)	5 (5)	1 (1)	1 (1)	3 (3)	3 (5)	1 (1)
Information on COVID-19 management	4 (3)	6 (7)	3 (3)	3 (4)	7 (7)	1 (1)	2 (2)	2 (2)	3 (4)	2 (2)
Support on exercise and diet	17 (13)	13 (14)	11 (12)	10 (15)	8 (8)	1 (1)	2 (2)	5 (6)	3 (4)	4 (4)
Support on exam preparation	23 (17)	30 (33)	31 (35)	33 (49)	32 (34)	7 (5)	12 (13)	9 (10)	9 (13)	8 (8)
Support on course material	24 (18)	30 (33)	30 (34)	34 (51)	25 (26)	7 (5)	9 (10)	10 (11)	13 (20)	10 (11)
Support with accommodation	-	-	-	-	-	0 (0)	0 (0)	0 (0)	0 (0)	0 (0)
Support with job applications/placements	-	-	-	-	-	1 (1)	4 (4)	3 (3)	3 (5)	7 (7)
Other	4 (3)	15 (16)	12 (14)	15 (23)	18 (19)	3 (2)	7 (8)	7 (8)	12 (18)	10 (11)

Some individuals reported in the free text box that whilst support had been provided by May, it was not needed. This number was quantified in the survey sent out in August, where 251 participants (*n* = 251/596, 42%) reported that they had not needed any support from their medical school to date. In August, 234 participants (*n* = 234/596, 39%) reported that they had received support that they needed, whilst 111 participants (*n* = 111/596, 19%) reported that they had not received the support they needed. The five most common areas of support that our participants needed and had not received from their medical schools by August were support with course material (*n* = 58/111, 52%), support with exam preparation (*n* = 50/111, 45%), online material/videos for self-support (*n* = 48/111, 43%), online face-to-face support (*n* = 26/111, 23%), and financial guidance (*n* = 24/111, 22%). Answers in the free text box highlighted that 9 participants felt that the support that they needed included a degree of clarity on plans for the next academic year. Table 4 details the support that medical students—by year group—wanted from their medical school in August.

### Support Provided to Participants by Foundation Schools

Of the 47 FiY1 doctors, 44 (*n* = 44/47, 94%) reported on the support that their foundation school had provided during both stages of this study. Over a third of the FiY1 doctors (*n* = 16/44, 36%) reported that their foundation school had provided some form of support by May. The three most common forms of support that individuals reported had been provided were information on COVID-19 symptoms (*n* = 10/44, 23%), information on COVID-19 management (*n* = 10/44, 23%) and online material or videos for self-support (*n* = 9/44, 20%). Two participants (*n* = 2/16, 13%) stated that the support provided had not been useful. Nearly half of the participants (*n* = 19/44, 43%) wanted more support from their foundation school. They principally wanted written support (*n* = 14/19, 74%), online face-to-face support (*n* = 7/19, 37%) and online material or videos for self-support (*n* = 6/19, 32%). All answers in the free text box related to greater clarity about their job role. Come August, only 7 FiY1 doctors (*n* = 7/44, 16%) reported needing further support. The three most common areas of support that our participants needed and had not received from their foundation schools by August were financial guidance (*n* = 4/7, 57%), online face-to-face support (*n* = 3/7, 43%) and support with placement (*n* = 2/7, 29%).

### Personal Protective Equipment Information and Provision

**Fig. 4:**
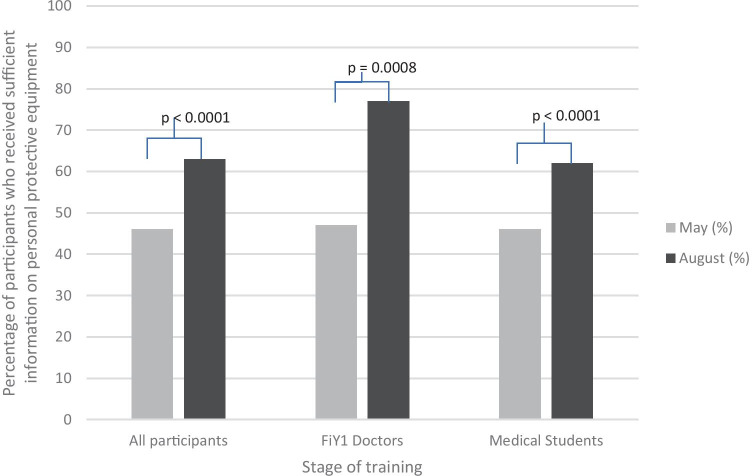
Percentage of participants who received sufficient information on personal protective equipment by stage of training. FiY1 interim foundation year 1

Overall, 534 participants (*n* = 534/640, 83%) provided data regarding the PPE information they had received in both May and August. There was a significant increase in the proportion of participants who felt they had received sufficient information on PPE between May (*n* = 245/534, 46%) and August (*n* = 338/534, 63%) (*p* < 0.0001) (Fig. 4). This significant increase was evident for both FiY1 doctors (May, 20/43, 47%; August, 33/43, 77%) and medical students (May, 225/491, 46%; August, 305/491, 62%). Among the 43 FiY1 doctors (*n* = 43/47, 91%) that reported on the PPE information they had received during both stages of this study, 38 provided data on whether they believed they had sufficient access to PPE. Thirty-three FiY1 doctors (*n* = 33/38, 87%) reported that they had sufficient access to PPE. Of the 5 FiY1 doctors (*n* = 5/38, 13%) that did not believe that they had sufficient access to PPE, 4 had also reported not having sufficient information on PPE. The 5 FiY1 doctors each reported a different reason for not having sufficient access to PPE: shortage, not offered by placement, changing PPE guidance, PPE available being uncomfortable to use, and insufficient training on using the PPE available.

### Infection Prevention and Control Training

**Fig. 5:**
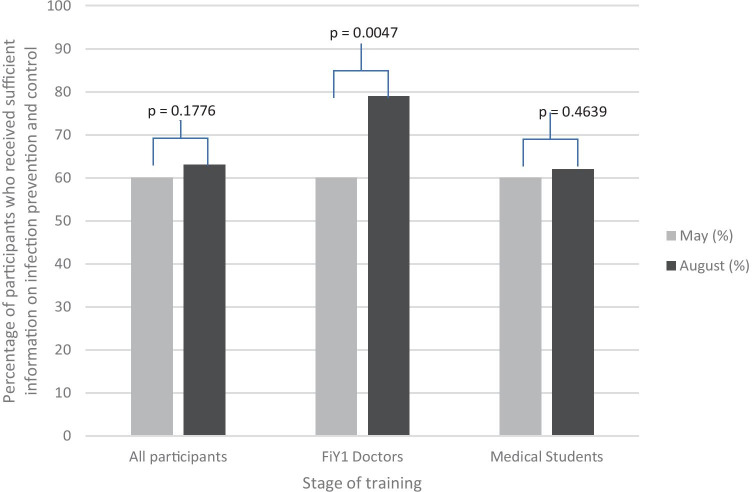
Percentage of participants who received sufficient training on infection prevention and control by stage of training. FiY1 interim foundation year 1

Overall, 535 participants (*n* = 535/640, 84%) provided data regarding the IPC training they had received in both May and August. There was an increase in the proportion of participants who felt they had received IPC training between May (*n* = 320/535, 60%) and August (*n* = 337/535, 63%) (*p* = 0.1776) (Fig. 5). This increase was evident for both FiY1 doctors (May, 26/43, 60%; August, 34/43, 79%) and medical students (May, 294/492, 60%; August, 303/492, 62%).

### Changes Experienced by Participants

The majority of participants (*n* = 581/640, 91%) detailed the areas of life that they felt the COVID-19 pandemic had impacted on positively and negatively during both stages of this study (Table 5). Nearly all areas of life surveyed were found to have negatively impacted more participants than positively impacted in both May and August. The exception was relationships with family and friends, where a greater number of individuals had been positively impacted than negatively impacted. Among the 579 participants (*n* = 579/640, 90%) that described—during both stages of this study—the areas of their education and career progression that they thought the COVID-19 pandemic would affect, clinical knowledge was the area most highly cited in both May and August (Table 6).

**Table 5:** Areas of life affected by COVID-19. These values do not total 100% as some participants did not report a positive or negative impact to that area of their life due to COVID-19

Areas of life	% Study total who report a positive impact		% Study total who report a negative impact	
	May (*n* = /581)	August (*n* = /581)	May (*n* = /581)	August (*n* = /581)
Studies	19 (111)	15 (90)	80 (467)	74 (428)
Social life	6 (36)	7 (40)	95 (552)	90 (523)
Vacations and travelling	0.3 (2)	2 (11)	86 (499)	87 (504)
Physical wellbeing	33 (193)	31 (182)	39 (226)	42 (243)
Future prospects	5 (28)	4 (26)	28 (164)	22 (128)
Research Involvement	7 (40)	8 (49)	21 (124)	23 (133)
Relationships with family and friends		53 (307)		39 (225)
Finances	24 (139)	26 (149)	29 (170)	26 (152)

**Table 6:** Areas of education and career progression that participants think the COVID-19 pandemic has or will affect

Areas of education and career progression	% Study total who think the COVID-19 pandemic will affect this area	
	May (*n* = /579)	August (*n* = /579)
Clinical knowledge	85 (491)	80 (466)
Anatomy knowledge	36 (206)	39 (224)
System based knowledge	34 (194)	39 (227)
Research opportunities	38 (226)	44 (256)
EPM scores	22 (125)	29 (169)
Public health knowledge	24 (138)	32 (184)
No areas	5 (29)	6 (37)

## Discussion

### Key Findings

Our findings show that nationally, medical students and FiY1 doctors reported that they had a lower mood than they did before the COVID-19 pandemic. However, their mood had improved in August compared to May. This may suggest that medical students and FiY1 doctors have started to adjust to the ‘new normal’ [34], thereby entering into the reconstruction phase of the pandemic [37]. It may also be due to the fact that more participants were receiving the support they needed in August compared to May. For instance, the proportion of medical students and FiY1 doctors reporting sufficiency of PPE information and IPC training increased by August, with a greater increase being evident in the sufficiency of PPE information.

In both May and August, the majority of individuals reported that ‘social distancing’, ‘reports on social media and news outlets’ and ‘self-isolation’ had had a negative impact on their mood. The exact same number of individuals (*n* = 424) continued to cite reports on social media and news outlets as having a negative influence on mood. Conversely, it is interesting to note that a smaller proportion of individuals cited social distancing (81 to 67%) and self-isolation (59 to 52%) to have had a negative impact on mood in August compared to May. It is likely that this came about due to the easing of restrictions [38]and the increased accessibility of a wider array of COVID-19 tests [39]. However, the majority of individuals continued to feel that their mood was being negatively affected by measures put in place to limit the spread of the virus. There were also factors that had a positive impact on mood for the majority of people in both May and August: more time at home/with family; reduced responsibility/ more free time; and them and their family members remaining unaffected from COVID-19. An interesting finding was that a smaller proportion of individuals cited ‘reduced responsibility/ more free time’ (59 to 54%) as having a positive impact on their mood in August compared to May, and similarly more people valued the positive impact on mood of spending ‘time away from work/university’ (46 to 56%) in August compared to May. Changing priorities during this period of time may have played a part. By August, all medical schools had started to re-open, which would have reduced free time available to students [40]. Similarly, the FiY1 doctors would also have been starting their new jobs as a FY1 doctors (US equivalent to PGY1), which would have been less supported than their prior job and perhaps more demanding. This was possibly mirrored in our findings that a greater proportion of individuals were citing the ‘strain of wearing protective equipment’ (9 to 17%) and ‘high demands in the work setting’ (9 to 12%) as having a negative influence on their mood in August compared to May.

### Implications

Given that approximately only three-fifths of medical students were reporting sufficiency in PPE provision and IPC training in August, there is a need for medical schools to provide students with adequate IPC training and comprehensive PPE education. This will help to prevent the virus spreading to patients as well as safeguard students, FiY1 doctors, their friends and their families. This should be mandatory before students are permitted to undergo clinical placements and could form part of summative assessment. However, given the necessity to maximise clinical exposure for students, medical schools should explore bridging options whilst their students undergo training. One such bridging option could be to create pre-recorded vignettes of clinical examinations of patients with normal and abnormal findings to allow students to learn and practise various clinical examinations [41]. This approach could be taken one step further to closer emulate the in-person experience: examinations conducted by doctors with real-life patients could be live fed to students. This has already been trialled for medical students at Imperial College London with virtual ward rounds, an experience one of the medical students deemed ‘invaluable’ [42]. In addition, in-person practising of communication skills and student-patient interactions could be substituted with online meetings with expert patients during the bridging period or through active involvement in virtual clinics [43, 44]. Given the ongoing nature of the pandemic, it is likely that many Objective Structured Clinical Examinations will be conducted online [45, 46]. As such, experience in taking histories and performing exams virtually may be the support with exam preparation that individuals have continued to perceive they need. It is important to note here that certain skills cannot be derived from only performing online simulations and watching videos. For example, safe donning and doffing of PPE requires psychomotor repetition to become competent [47]. In-person teaching may be key for individuals to be adequately trained to wear PPE.

In addition to providing support on PPE, there is a need for other types of support. In both May and August, online face-to-face support and online material or videos for self-support were two of the top five requests for support made by our participants. There have been reports that twice as many doctors and medical students have been seeking support for their mental health since the onset of the pandemic, and this has partly been due to feeling unsupported [48]and potentially due to being more directly exposed to deaths caused by SARS-CoV-2 [48, 49]. It is important to address this by creating confidential in-person or online spaces where medical students and doctors can talk about the impact of their work on their mental and physical health. Creating mini groups for this purpose could empower individuals to not only share their experiences but gain and provide support to each other, for example by encouraging individuals to keep physically active or motivating individuals to do activities that they find fulfilling [50].

An important and promising finding was that fewer individuals were worried about catching COVID-19 in August compared to May. This is likely to be due to the reduction in daily COVID-19 cases and deaths between May and August [51], which was reported by a sizeable proportion of our study population to have had a positive impact on mood. It could also suggest that improvements in personal protect equipment (PPE) provision and infection prevention control (IPC) since May [19]have allayed concerns among our participants. Alternatively, it may suggest that many individuals working in healthcare have already been affected by COVID-19 [52], and therefore believe they are protected from getting infected again. If it is due to the latter reason, it will be important for medical schools and employers to emphasise that there is a risk of reinfection by SARS-CoV-2 [53, 54]. Therefore, it remains important for individuals to be vigilant and continue following IPC protocols even if they have previously been infected. This may be particularly challenging given that an increasing number of individuals are citing the strain of wearing PPE as having a negative impact on their mood. To combat this, there needs to be a concerted effort to create comfortable PPE [55], and make that accessible to all medical staff, including medical students and FiY1 doctors. Given that individuals from ethnic minority backgrounds continued to remain anxious about catching COVID-19, there also needs to be a greater focus on ensuring sufficiency of PPE information and IPC training from the perspective of students from these demographic backgrounds.

It is also important to register that in both May and August, ‘more time at home/with family’ was the factor that the largest number of individuals identified as having a positive impact on mood. This has important connotations for international students, many of whom have been unable to get back to their family homes [56]. As such, it is crucial that medical schools act as a substitute source of support and ensure that their students feel provided for and safe. Our recommendations regarding support that can be offered by medical schools can be based on data pertaining to ‘further support sought’ by our participants. The primary support sought was regarding exam preparation and support with course material. With the substantial impact on in person teaching, a robust online infrastructure to deliver teaching and revision material should be implemented and where possible built upon for posterity. Multiple lessons have been learnt in the field of technology-enhanced learning during this pandemic [57, 58], and this new knowledge should be utilised to create a bank of long-term engaging and educational tasks that can be implemented online; this should especially be considered for education involving medical imaging [59]. Additionally, universities should prioritise providing their students with up-to-date information regarding assessments as well as further possible changes to these, during the on-going pandemic. Creating these communication streams now may also prove beneficial to future generations of students, as lack of timely communication is often cited as a cause of dissatisfaction among medical professionals [60]. Financial guidance was sought by participants, and we recommend that medical schools support schemes including maintenance grants, accommodation fee deferral and paid work for medical students. This may also prove beneficial in the long-term for widening access initiatives.

In addition, given families are a potential source of help for many of our participants, it is vital to alleviate any issues that prevent students from accessing this support, such as building timetables that enable visits home and self-isolation time. Therefore, it is worthy of note that the majority of participants—particularly students from ethnic minority backgrounds (*n* = 125)—in both May and August have been worried about transmitting the infection, and their friends and family catching COVID-19. To mitigate becoming a vector for onward transmission, students and FiY1 doctors may have chosen to self-isolate [61]. In doing so, they may be less connected with their normal support structures, thereby increasing their risk of depression and anxiety [62, 63]. Consequently, there needs to be a concerted effort to reduce the anxiety of transmitting the infection to family. One strategy could be actively promoting the fact that published studies have not found that social distancing reduces the risk of asymptomatic healthcare workers transmitting COVID-19 infection to other household members [64], and that healthcare workers are not one of the main transmission risks for relatives [65]. An alternative strategy of reassurance may have been implemented in 2021, as medical students were regularly tested and prioritised for vaccination [66]. Test results could have allayed fears of individuals being an unintentional vector for the disease. A study is currently being run to elucidate the impact these schemes have had on student’s anxiety levels. It is also worth exploring how the language and narrative used by the media may have played into this anxiety, especially early on during the pandemic [67].

Our findings show that ‘reports on social media and news outlets’ have had a negative influence on mood for three-quarters of our participants for four months. This necessitates action. Simply advocating for news outlets to focus on health promoting behaviours instead of reporting negative news stories will not sufficiently tackle this issue. This is because of the inherent tendency of individuals to be more attentive to negative news content [68]. Of significance, however, is the fact that misinformation and conspiracy theories present on social media have been linked to an increase in anxiety and Sinophobia [69]. One way to address this would be for social media providers to introduce a trust rating for news reports that crowdsources people’s judgement on the reliability and accuracy of the information; this in turn could be used by ranking algorithms to minimise the number of people exposed to potentially untrustworthy information [70].

### Limitations

There are key limitations to be noted when interpreting the findings of this study. The principal issue is that people vary in their ability to recognise their own needs and have different comfort levels of asking for help. Therefore, there is a risk that we are not capturing the true needs of our population. However, it should be noted that reluctance to seek help can be overcome through interactions by a caring person, who has the correct information and encourages the seeking of assistance [37]. This is the role that medical schools need to play. Moreover, our findings on IPC training and PPE information reflect both the training received and each individual’s perception of that training, and it would be of significant interest to conduct research to disentangle these perceptions. Additionally, our data collection did not allow us to determine the extent to which each factor affected mood. It is unclear whether certain factors had more or less of an impact on the mental health of students and whether this changed as the pandemic progressed. Also given the lack of baseline data from a pre-COVID era, it is unclear whether certain factors would have impacted students similarly if the COVID-19 pandemic had not occurred. Finally, our study included very few FiY1 doctors and ethnic minority medical students, and therefore may not be representative of the population of FiY1 doctors or the population of ethnic minority medical students.

## Conclusion

Our prospective study design enabled the dynamic ascertainment of participants as they lived through the COVID-19 pandemic, making the data especially pertinent. Widescale participation of medical students across the UK has allowed for the effects of the pandemic to be studied on a national scale, with statistically significant results. Ultimately, our findings have actionable implications that can better protect medical students and FiY1 doctors as they acclimatise to a working environment that has been radically changed by COVID-19. It is critical that mental health is put at the very centre of recovery plans, and there is an emphasis on support.

## Electronic supplementary material

Below is the link to the electronic supplementary material.

Supplementary file1 (PDF 2959 kb)

Supplementary file1 (PDF 2635 kb)

Supplementary file1 (DOCX 128 kb)
