# Supplementary material for: SPICE-19: a 3-Month Prospective Cohort Study of 640 Medical Students and Foundation Doctors
Source: Med Sci Educ. 2021 Jul 21;31(5):1621–37. doi: 10.1007/s40670-021-01349-0 (PMC8294310; doi:10.1007/s40670-021-01349-0)
Supplement: Supplementary file 1 — Supplementary file1 (PDF 2959 kb) [file 40670_2021_1349_MOESM1_ESM.pdf]

Default Question Block

Social and Psychological Impact of COVID-19 on medical students: a national survey Evaluation (SPICE-19)

Ethics Approval Reference R69297/RE001

General Information
The aim of this study is to identify the impact of the COVID-19 pandemic on the social and psychological wellbeing of medical students and interim foundation doctors in the United Kingdom (UK). We would like to invite medical students across all medical schools of the UK to participate in the SPICE-19 study to evaluate the effects the novel coronavirus (COVID-19) pandemic has had on their mental health. Please read the following information before deciding to participate, and contact the research team at soham.bandyopadhyay@st-hildas.ox.ac.uk if you have any questions.

What is the purpose of this study?
The purpose of this study is to evaluate potential associations between the COVID-19 pandemic and the social and psychological wellbeing of medical students. This study also seeks to identify policies introduced by medical schools that were successful in supporting medical students and interim foundation doctors..

Why have I been invited to take part?
You are being invited to participate in this study because we are recruiting medical students and interim foundation doctors from across the UK, to **provide information on the psychological and social wellbeing**, before and during the COVID-19 pandemic. You are eligible to participate if you are a medical student or interim foundation doctor within the UK (England, Northern Ireland, Scotland and Wales) and have access to the internet.

What happens if I choose to participate?
If you choose to participate in this voluntary survey, you will be asked to complete a questionnaire about your background, the positive and negative impacts of the COVID-19 pandemic on your mental health, and the support offered to you. This study is voluntary. If you decide not to participate this will not impact your academic standing in any way. If you decide to take part, you will be asked to complete the survey by clicking on the link below. A follow-up survey will be sent by an email link in August. Each survey is expected to take about 10 -15 minutes to complete – but there is no time limit and you can take as much time as you like. No background knowledge is required.

We will ask for your consent for the collection and storage of data in accordance with the General Data Protection Regulation (GDPR) within the survey. For more information on GDPR please click on the following link: https://gdpr-info.eu/.

Do I have to participate?
Please note that your participation is voluntary. You may withdraw at any point during the questionnaire for any reason, before submitting your answers, by closing the browser. If you wish to discontinue from this study, you are free to do so at any time by not participating in any future questionnaires. In cases of withdrawal, no new data will be collected or linked to other data from that point on. If you do not want to answer some of the questions you do not have to, but you can still be in the study. As all questions are optional, we have included a 'Prefer not to answer' option for each set of questions. Your decision whether or not to be part of the study will not affect your academic standing or your access to university support services.

Are there any possible risks involved with my participation?
Some of the questions that we ask may cause upset or bring up painful memories. If you experience any distress from participating in this study, you may stop the survey at any time or skip any upsetting questions. If your distress continues after leaving the survey, we have provided a list of supportive services nationwide that can be helpful and that you might consider contacting.

What are the possible benefits of participating?
Despite not have any immediate individual benefits by participating in this survey, you are given the opportunity to contribute to valuable and innovative research which could be used in the future by medical universities and the world. You may find this survey an opportunity to self-reflect on the current events and anonymously express your feelings related to this global event and the impact this has on your medical career.

How will my data be used?
Your answers will be completely anonymous, and we will take all reasonable measures to ensure that they remain confidential. Your data will be stored in a password-protected file and may be used in academic publications. Your IP address will not be stored. If you provide us with your email address, we will delete that information at the end of the study. No answers will be linked to your email address. Research data – your anonymised answers – will be stored for a minimum of ten years after publication or public release.

Who will have access to my data?
Qualtrics is the data controller with respect to the personal data they hold about you and, as such, will determine how your personal data is used. Please see their privacy notice here: https://www.qualtrics.com/privacy-statement. Qualtrics will share any email address you provide and your anonymised answers with the University of Oxford, for the purposes of research.
Responsible members of the University of Oxford and funders may be given access to data for monitoring and/or audit of the study to ensure we are complying with guidelines, or as otherwise required by law.

Will the research be published?
The findings of the study may be published in peer reviewed journals, presented at relevant conferences and meetings and a summary of the findings will be made available on the website.

Who do I contact if I have a concern about the study or I wish to complain?
If you have a concern about any aspect of this project, please speak to the researcher Soham Bandyopadhyay on soham.bandyopadhyay@st-hildas.ox.ac.uk who will do his best to answer your query. The researchers should acknowledge your concern within 10 working days and give you an indication of how they intend to deal with it. If you remain unhappy or wish to make a formal complaint, please contact the Chair of the Medical Sciences Inter-Divisional Research Ethics Committee: Email: ethics@medsci.ox.ac.uk; Address: Research Services, University of Oxford, Wellington Square, Oxford OX1 2JD OR

The Chair will seek to resolve the matter in a reasonably expeditious manner.

How do I find out what was learned in this study?
This study is expected to be completed by approximately September 2020. If you would like a brief summary of the results, please write to us by email to request information

Who to contact for further details?
For any further questions or more information on the study, please contact us on the following email address: soham.bandyopadhyay@st-hildas.ox.ac.uk. Alternatively, you could contact principal investigator Prof Kate Saunders at kate.saunders@psych.ox.ac.uk.

Please note that you may only participate in this survey if you are 18 years of age or over.

○ I certify that I am 18 years of age or over

If you have read the information above and agree to participate with the understanding that the data (including any personal data) you submit will be processed accordingly, please check the relevant box below to get started.

○ Yes, I agree to take part

Tick this box if you are willing to take part in the follow-up survey and are happy to be contacted via your email address

○ Yes, I agree to be contacted for a follow-up study (please enter a valid email address below)

Block 1

1. BACKGROUND INFORMATION

Are you a medical student or an interim Foundation Year 1 doctor?

○ Medical student

○ Interim Foundation Doctor

Which medical school are you currently enrolled in?

Which medical school were you previously enrolled in?

How many (if any) years of medical school (excluding intercalation) have you completed so far?

Are you currently undertaking you intercalated degree?

- ○ Yes
- ○ No

Have you previously undertaken an intercalated degree?

- ○ Yes
- ○ No

How old are you?

What is your gender?

- ○ Male
- ○ Female
- ○ Non-binary
- ○ Prefer to self-describe (please specify)
- ○ Prefer not to answer

What is your ethnicity?

- ○ English/Welsh/Scottish/Northern Irish/British

- ○ Irish
- ○ Gypsy or Irish Traveller
- ○ Any other White background, please write in
- ○ White and Black Caribbean
- ○ White and Black African
- ○ White and Asian
- ○ Any other Mixed/Multiple ethnic background, please write in
- ○ Indian
- ○ Pakistani
- ○ Bangladeshi
- ○ Chinese
- ○ Any other Asian background, please write in
- ○ African
- ○ Caribbean
- ○ Any other Black/African/Caribbean background, please write in
- ○ Other ethnic group, please write in
- ○ Prefer not to say

Block 2

2. Wellbeing

The following questions ask about how you are currently feeling during the COVID-19 outbreak. Please answer the extent to which you agree with each statement.

	Strongly disagree	Disagree	Somewhat disagree	Neither agree nor disagree	Somewhat agree	Agree	Strongly agree

	Strongly disagree	Disagree	Somewhat disagree	Neither agree nor disagree	Somewhat agree	Agree	Strongly agree
I think COVID-19 is a very serious issue	○	○	○	○	○	○	○
I am worried that I will catch COVID-19	○	○	○	○	○	○	○
I am worried that friends and family will catch COVID-19	○	○	○	○	○	○	○
I am afraid to leave the house right now	○	○	○	○	○	○	○
I am worried I might transmit the infection to someone else	○	○	○	○	○	○	○
I am worried we won't have enough food and other essential items during the outbreak	○	○	○	○	○	○	○
I am worried about the amount of money we have coming in	○	○	○	○	○	○	○
I am worried about the long-term impact this will have on my job prospects and the economy	○	○	○	○	○	○	○

How would you rate your mood before COVID-19 affected the UK? 0 being the worst mood you can imagine and 100 being the best mood you can imagine

	Worst										Best
	0	10	20	30	40	50	60	70	80	90	100
Rate your mood by moving the slider											

How would you rate your mood now? 0 being the worst mood you can imagine and 100 being the best mood you can imagine

	Worst										Best
	0	10	20	30	40	50	60	70	80	90	100
Rate your mood by moving the slider											

Has your mood been negatively affected by any of the following? Please select all that apply.

- ☐ Reports on social media and news outlets
- ☐ Self-isolation
- ☐ Social distancing
- ☐ Getting infected
- ☐ Relatives or friends getting infected
- ☐ Recent bereavement of someone you know from COVID-19
- ☐ Financial worries
- ☐ Career uncertainty
- ☐ Elective cancelled
- ☐ Holiday cancelled
- ☐ Social exclusion
- ☐ Stigmatisation
- ☐ Physician strain of wearing protective equipment
- ☐ High demands in the work setting
- ☐ Deterioration of physical health
- ☐ Reduction in research opportunities
- ☐ Prefer not to answer
- ☐ None of the above

☐ Other (please state)

Has your mood been positively affected by any of the following? Please select all that apply.

☐ More time at home/ with family

☐ Time away from work/university

☐ Reduced responsibilities/ more free time

☐ Online learning

☐ Cancelled/ Open book exams

☐ You and your family members remained unaffected from COVID19

☐ Improved financial status

☐ Volunteering opportunities

☐ Opportunities for paid work

☐ Improvements in physical health

☐ Increase in research opportunities

☐ Prefer not to answer

☐ None of the above

☐ Other (please state)

Below are some emotions. Please tick the box that best describes the extent to which you are currently experiencing each emotion.

	Always	Most of the time	About half the time	Sometimes	Never
Anxious	○	○	○	○	○
Elated	○	○	○	○	○
Sad	○	○	○	○	○
Irritable	○	○	○	○	○
Energetic	○	○	○	○	○
Cheerful	○	○	○	○	○
Guilt	○	○	○	○	○
Shame	○	○	○	○	○

	Always	Most of the time	About half the time	Sometimes	Never
Self-doubt	○	○	○	○	○

Have you faced any social stigma or discrimination associated with COVID-19?

○ Yes

○ No

○ Prefer not to answer

What stigma or discrimination did you experience?

Which of the following have you used to help with your mental wellbeing? Please select all that apply.

☐ Exercise

☐ Cooking/ baking

☐ Gardening

☐ Listening to music

☐ Playing music

☐ Artwork

☐ Gaming

☐ Watching movies or TV series

☐ Reading books

☐ Staying connected with friends/relatives using online tools

☐ Meditation

☐ Mental health apps

☐ Prefer not to answer

☐ None of the above

☐ Other (please state)

Block 3

3. SUPPORT

Has your university provided any form of support?

- ○ Yes
- ○ No

What support has the university provided you with? Please select all that apply

- ☐ Online material/videos for self-support
- ☐ Online face-to-face support
- ☐ Letters, emails, or any other form of written support
- ☐ Financial guidance
- ☐ Information on COVID-19 symptoms
- ☐ Information on COVID-19 management
- ☐ Support on exercise and diet
- ☐ Support on exam preparation
- ☐ Support on course material
- ☐ Other (please state) ________

Has your university support been useful?

- ○ Yes
- ○ No

Why not?

____

Would you like your university to provide you with more support?

- ○ Yes
- ○ No

Which of the following would be useful for your university to provide? Please select all that apply

- ☐ Online material/videos for self-support
- ☐ Online face-to-face support
- ☐ Letters, emails, or any other form of written support
- ☐ Financial guidance
- ☐ Information on COVID-19 symptoms
- ☐ Information on COVID-19 management
- ☐ Support on exercise and diet
- ☐ Support on exam preparation
- ☐ Support on course material
- ☐ Other (please state) ______

Has your Foundation School provided any form of support?

- ○ Yes
- ○ No

What support have they provided you with? Please select all that apply

- ☐ Online material/videos for self-support
- ☐ Online face-to-face support
- ☐ Letters, emails, or any other form of written support
- ☐ Financial guidance
- ☐ Information on COVID-19 symptoms
- ☐ Information on COVID-19 management
- ☐ Support on exercise and diet

- ☐ Support on exam preparation
- ☐ Support on course material
- ☐ Other (please state) ________

Has the support been useful?

- ○ Yes
- ○ No

Why not?

____

Would you like your Foundation School to provide you with more support?

- ○ Yes
- ○ No

Which of the following would it have been useful for them to provide? Please select all that apply

- ☐ Online material/videos for self-support
- ☐ Online face-to-face support
- ☐ Letters, emails, or any other form of written support
- ☐ Financial guidance
- ☐ Information on COVID-19 symptoms
- ☐ Information on COVID-19 management
- ☐ Support on exercise and diet
- ☐ Support on exam preparation
- ☐ Support on course material
- ☐ Other (please state) ________

Do you believe that you have been provided with sufficient information on Personal Protective Equipment (PPE)?

- ○ Yes
- ○ No

Who has provided this information?

- ☐ University
- ☐ NHS Trust
- ☐ A national body e.g. Health Education England
- ☐ Other (please state)

Do you believe that you have been provided with sufficient training on infection prevention and control?

- ○ Yes
- ○ No

Who has provided this training?

- ☐ University
- ☐ NHS Trust
- ☐ A national body e.g. Health Education England
- ☐ Other (please state)

Block 4

4. Changes resulting from COVID-19

Which areas of your life do you feel that COVID-19 has impacted on negatively? Please select all that apply

- ☐ Studies

- ☐ Social life
- ☐ Vacations and travelling
- ☐ Physical well being
- ☐ Future prospects
- ☐ Research Involvement
- ☐ Financially
- ☐ No areas
- ☐ Prefer not to answer
- ☐ Other (please state)

Which areas of your life do you feel that COVID-19 has impacted on positively? Please select all that apply

- ☐ Studies
- ☐ Social life
- ☐ Vacations and travelling
- ☐ Physical well being
- ☐ Future prospects
- ☐ Research Involvement
- ☐ Financially
- ☐ No areas
- ☐ Prefer not to answer
- ☐ Other (please state)

Which of these emotions are you experiencing with regards to becoming a new doctor during the COVID-19 pandemic?

	Always	Most of the time	About half the time	Sometimes	Never
Anxious	○	○	○	○	○
Elated	○	○	○	○	○
Sad	○	○	○	○	○

	Always	Most of the time	About half the time	Sometimes	Never
Irritable	○	○	○	○	○
Energetic	○	○	○	○	○
Cheerful	○	○	○	○	○
Guilt	○	○	○	○	○
Shame	○	○	○	○	○
Other (please specify)	○	○	○	○	○

Are you still getting teaching from your medical school?

- ○ Yes
- ○ No

Are you satisfied with the quality and quantity of teaching being provided?

- ○ Yes
- ○ No

Why not?

How have the changes to medical school teaching affected you?

- ○ Positively
- ○ Negatively
- ○ No effect
- ○ Prefer not to answer

Which of the following areas of your education and career progression do you think the COVID-19 pandemic has or will affect? Please select all that apply

- ☐ Clinical knowledge

- [ ] Anatomy knowledge
- [ ] Systems based knowledge
- [ ] Research opportunities
- [ ] EPM scores
- [ ] Public health knowledge
- [ ] No areas
- [ ] Prefer not to answer
- [ ] Other (please state)

Have you worked or are you currently working within a healthcare setting during the course of this pandemic?

- ○ Yes
- ○ No

Which of the following roles have you taken up whilst working in the hospital?

- [ ] Full time doctor
- [ ] Part time doctor
- [ ] Locum doctor
- [ ] Administrative role
- [ ] Nursing role
- [ ] Healthcare Assistant
- [ ] Receptionist
- [ ] Clinical technician
- [ ] Porter
- [ ] Prefer not to answer
- [ ] Other (please state)

What are the reasons for this?

- ☐ Not enough opportunities
- ☐ Risk of getting infection
- ☐ Risk of passing infection to family members
- ☐ Not interested in getting a job yet
- ☐ Caregiver role
- ☐ Childcare responsibility
- ☐ Underlying medical condition
- ☐ Prefer not to answer
- ☐ Other (please state) ____________

How likely are you to continue in a career in medicine?

- ○ Extremely likely
- ○ Somewhat likely
- ○ Neither likely nor unlikely
- ○ Somewhat unlikely
- ○ Extremely unlikely

Thank you for completing the survey.

Survey Powered By Qualtrics
