# Supplementary material for: SPICE-19: a 3-Month Prospective Cohort Study of 640 Medical Students and Foundation Doctors
Source: Med Sci Educ. 2021 Jul 21;31(5):1621–37. doi: 10.1007/s40670-021-01349-0 (PMC8294310; doi:10.1007/s40670-021-01349-0)
Supplement: Supplementary file 2 — Supplementary file1 (PDF 2635 kb) [file 40670_2021_1349_MOESM2_ESM.pdf]

Default Question Block

Social and Psychological Impact of COVID-19 on medical students: a national survey Evaluation (SPICE-19)
FOLLOW UP SURVEY

Ethics Approval Reference R69297/RE001

General Information
The aim of this study is to identify the impact of the COVID-19 pandemic on the social and psychological wellbeing of medical students and interim foundation doctors in the United Kingdom (UK). We would like to invite medical students across all medical schools of the UK to participate in the SPICE-19 study to evaluate the effects the novel coronavirus (COVID-19) pandemic has had on their mental health. Please read the following information before deciding to participate, and contact the research team at soham.bandyopadhyay@st-hildas.ox.ac.uk if you have any questions.

What is the purpose of this study?
The purpose of this study is to evaluate potential associations between the COVID-19 pandemic and the social and psychological wellbeing of medical students. This study also seeks to identify policies introduced by medical schools that were successful in supporting medical students and interim foundation doctors..

Why have I been invited to take part?
You are being invited to participate in this study because we are recruiting medical students and interim foundation doctors from across the UK, to provide information on their psychological and social wellbeing, before and during the COVID-19 pandemic. You are eligible to participate if you are a medical student or interim foundation doctor within the UK (England, Northern Ireland, Scotland and Wales) and have access to the internet.

What happens if I choose to participate?
If you choose to participate in this voluntary survey, you will be asked to complete a questionnaire about your background, the positive and negative impacts of the COVID-19 pandemic on your mental health, and the support offered to you. This study is voluntary. If you decide not to participate this will not impact your academic standing in any way. If you decide to take part, you will be asked to complete the survey by clicking on the link below. A follow-up survey will be sent by an email link in August. Each survey is expected to take about 10 -15 minutes to complete – but there is no time limit and you can take as much time as you like. No background knowledge is required.

We will ask for your consent for the collection and storage of data in accordance with the General Data Protection Regulation (GDPR) within the survey. For more information on GDPR please click on the following link: https://gdpr-info.eu/.

Do I have to participate?
Please note that your participation is voluntary. You may withdraw at any point during the questionnaire for any reason, before submitting your answers, by closing the browser. If you wish to discontinue from this study, you are free to do so at any time by not participating in any future questionnaires. In cases of withdrawal, no new data will be collected or linked to other data from that point on. If you do not want to answer some of the questions you do not have to, but you can still be in the study. As all questions are optional, we have included a 'Prefer not to answer' option for each set of questions. Your decision whether or not to be part of the study will not affect your academic standing or your access to university support services.

Are there any possible risks involved with my participation?
Some of the questions that we ask may cause upset or bring up painful memories. If you experience any distress from participating in this study, you may stop the survey at any time or skip any upsetting questions. If your distress continues after leaving the survey, we have provided a list of supportive services nationwide that can be helpful and that you might consider contacting.

What are the possible benefits of participating?
Despite not have any immediate individual benefits by participating in this survey, you are given the opportunity to contribute to valuable and innovative research which could be used in the future by medical universities and the world. You may find this survey an opportunity to self-reflect on the current events and anonymously express your feelings related to this global event and the impact this has on your medical career.

How will my data be used?
Your answers will be completely anonymous, and we will take all reasonable measures to ensure that they remain confidential. Your data will be stored in a password-protected file and may be used in academic publications. Your IP address will not be stored. If you provide us with your email address, we will delete that information at the end of the study. No answers will be linked to your email address. Research data – your anonymised answers – will be stored for a minimum of ten years after publication or public release.

Who will have access to my data?
Qualtrics is the data controller with respect to the personal data they hold about you and, as such, will determine how your personal data is used. Please see their privacy notice here: https://www.qualtrics.com/privacy-statement. Qualtrics will share any email address you provide and your anonymised answers with the University of Oxford, for the purposes of research.
Responsible members of the University of Oxford and funders may be given access to data for monitoring and/or audit of the study to ensure we are complying with guidelines, or as otherwise required by law.

Will the research be published?
The findings of the study may be published in peer reviewed journals, presented at relevant conferences and meetings and a summary of the findings will be made available on the website.

Who do I contact if I have a concern about the study or I wish to complain?
If you have a concern about any aspect of this project, please speak to the researcher Soham Bandyopadhyay on soham.bandyopadhyay@st-hildas.ox.ac.uk who will do his best to answer your query. The researchers should acknowledge your concern within 10 working days and give you an indication of how they intend to deal with it. If you remain unhappy or wish to make a formal complaint, please contact the Chair of the Medical Sciences Inter-Divisional Research Ethics Committee: Email: ethics@medsci.ox.ac.uk; Address: Research Services, University of Oxford, Wellington Square, Oxford OX1 2JD OR

The Chair will seek to resolve the matter in a reasonably expeditious manner.

How do I find out what was learned in this study?
This study is expected to be completed by approximately September 2020. If you would like a brief summary of the results, please write to us by email to request information

Who to contact for further details?
For any further questions or more information on the study, please contact us on the following email address: soham.bandyopadhyay@st-hildas.ox.ac.uk. Alternatively, you could contact principal investigator Prof Kate Saunders at kate.saunders@psych.ox.ac.uk

If you have read the information above and agree to participate with the understanding that the data (including any personal data) you submit will be processed accordingly, please check the relevant box below to get started.

○ Yes, I agree to take part

Please write the email id the questionnaire was sent to:

Block 2

1. Wellbeing

The following questions ask about how you are currently feeling during the COVID-19 outbreak. Please answer the extent to which you agree with each statement.

	Strongly disagree	Disagree	Neither agree nor disagree	Agree	Strongly agree
I am worried that I will catch COVID-19	○	○	○	○	○
I am worried that friends and family will catch COVID-19	○	○	○	○	○
I am afraid to leave the house right now	○	○	○	○	○
I am worried I might transmit the infection to someone else	○	○	○	○	○
I am worried about missing school/ work	○	○	○	○	○

	Strongly disagree	Disagree	Neither agree nor disagree	Agree	Strongly agree
I am worried about the amount of money we have coming in	○	○	○	○	○
I am worried about the long-term impact this will have on my job prospects and the economy	○	○	○	○	○

How would you rate your mood now? 0 being the worst mood you can imagine and 100 being the best mood you can imagine

	Worst										Best
	0	10	20	30	40	50	60	70	80	90	100
Rate your mood by moving the slider											

Has your mood been negatively affected by any of the following over the last 2 months? Please select all that apply.

- ☐ Reports on social media and news outlets
- ☐ Self-isolation
- ☐ Social distancing
- ☐ Getting infected
- ☐ Relatives or friends getting infected
- ☐ Recent bereavement of someone you know from COVID-19
- ☐ Financial worries
- ☐ Career uncertainty
- ☐ Elective cancelled
- ☐ Holiday cancelled
- ☐ Social exclusion

- ☐ Stigmatisation
- ☐ Physician strain of wearing protective equipment
- ☐ High demands in the work setting
- ☐ Deterioration of physical health
- ☐ Reduction in research opportunities
- ☐ Uncertainty related to medical education
- ☐ Changes to medical education
- ☐ Uncertainty related to examinations
- ☐ Prefer not to answer
- ☐ None of the above
- ☐ Other (please state)

Has your mood been positively affected by any of the following over the last 2 months? Please select all that apply.

- ☐ More time at home/ with family
- ☐ Time away from work/university
- ☐ Reduced responsibilities/ more free time
- ☐ Online learning
- ☐ Cancelled/ Open book exams
- ☐ You and your family members remained unaffected from COVID19
- ☐ Improved financial status
- ☐ Volunteering opportunities
- ☐ Opportunities for paid work
- ☐ Improvements in physical health
- ☐ Increase in research opportunities
- ☐ More medical school teaching
- ☐ Return to face-to-face teaching
- ☐ Reduction in COVID-19 cases in the UK
- ☐ Relaxation of measures imposed to control the spread of COVID-19

☐ Prefer not to answer

☐ None of the above

☐ Other (please state)

Below are some statements about feelings and thoughts. Please choose the box that best describes your experience of each over the last 2 weeks.

	Always	Most of the time	About half the time	Sometimes	Never
I've been feeling optimistic about the future	○	○	○	○	○
I've been feeling useful	○	○	○	○	○
I've been feeling relaxed	○	○	○	○	○
I've been feeling interested in other people	○	○	○	○	○
I've had energy to spare	○	○	○	○	○
I've been dealing with problems well	○	○	○	○	○
I've been thinking clearly	○	○	○	○	○
I've been feeling good about myself	○	○	○	○	○
I've been feeling close to other people	○	○	○	○	○
I've been feeling confident	○	○	○	○	○
I've been able to make up my own mind about things	○	○	○	○	○
I've been feeling loved	○	○	○	○	○
I've been interested in new things	○	○	○	○	○
I've been feeling cheerful	○	○	○	○	○

Block 3

2. SUPPORT

Over the last two months has your university provided you with support that you needed?

- ○ Yes
- ○ No
- ○ No support needed

What support has the university provided you with? Please select all that apply

- ☐ Online material/videos for self-support
- ☐ Online face-to-face support
- ☐ Letters, emails, or any other form of written support
- ☐ Financial guidance
- ☐ Information on COVID-19 symptoms
- ☐ Information on COVID-19 management
- ☐ Support on exercise and diet
- ☐ Support on exam preparation
- ☐ Support on course material
- ☐ Support with accommodation
- ☐ Support with job applications/ placements
- ☐ Other (please state) ____________

What support did you need your university to provide you with?

- ☐ Online material/videos for self-support
- ☐ Online face-to-face support
- ☐ Letters, emails, or any other form of written support
- ☐ Financial guidance

- ☐ Information on COVID-19 symptoms
- ☐ Information on COVID-19 management
- ☐ Support on exercise and diet
- ☐ Support on exam preparation
- ☐ Support on course material
- ☐ Support with accommodation
- ☐ Support with job applications/ placements
- ☐ Other (please state) ________

Over the last two months has your Foundation School provided you with support that you needed?

- ○ Yes
- ○ No
- ○ No support needed

What support did you need your Foundation School to provide you with?

- ☐ Online material/videos for self-support
- ☐ Online face-to-face support
- ☐ Letters, emails, or any other form of written support
- ☐ Financial guidance
- ☐ Information on COVID-19 symptoms
- ☐ Information on COVID-19 management
- ☐ Support on exercise and diet
- ☐ Support on exam preparation
- ☐ Support on course material
- ☐ Support with accommodation
- ☐ Support with job applications/ placements
- ☐ Other (please state) ________

What support have they provided you with? Please select all that apply

- ☐ Online material/videos for self-support
- ☐ Online face-to-face support
- ☐ Letters, emails, or any other form of written support
- ☐ Financial guidance
- ☐ Information on COVID-19 symptoms
- ☐ Information on COVID-19 management
- ☐ Support on exercise and diet
- ☐ Support on exam preparation
- ☐ Support on course material
- ☐ Support with accommodation
- ☐ Support with job applications/ placements
- ☐ Other (please state) ________

Do you believe that you have been provided with sufficient information on Personal Protective Equipment (PPE)?

- ○ Yes
- ○ No

Who has provided this information?

- ☐ University
- ☐ NHS Trust
- ☐ A national body e.g. Health Education England
- ☐ Other (please state) ________

Do you believe that you have had sufficient access to PPE during your placement(s)?

- ○ Yes
- ○ No
- ○ Not applicable

Why did you not have sufficient access to PPE during your placement(s)?

- ○ Shortages of PPE
- ○ ii. Uncomfortable to use
- ○ Not trained on how to use PPE
- ○ Not offered by placement(s)
- ○ Other

Do you believe that you have been provided with sufficient training on infection prevention and control?

- ○ Yes
- ○ No

Who has provided this training?

- ☐ University
- ☐ NHS Trust
- ☐ A national body e.g. Health Education England
- ☐ Other (please state)

Block 4

3. Changes resulting from COVID-19

Which areas of your life do you feel that COVID-19 has impacted on negatively over the last 2 months? Please select all that apply

- ☐ Studies
- ☐ Social life
- ☐ Vacations and travelling
- ☐ Physical well being
- ☐ Future prospects
- ☐ Research Involvement
- ☐ Finances
- ☐ Relationships with family and friends
- ☐ No areas
- ☐ Prefer not to answer
- ☐ Other (please state)

Which areas of your life do you feel that COVID-19 has impacted on positively over the last 2 months? Please select all that apply

- ☐ Studies
- ☐ Social life
- ☐ Vacations and travelling
- ☐ Physical well being
- ☐ Future prospects
- ☐ Research Involvement
- ☐ Finances
- ☐ Relationships with family and friends
- ☐ No areas
- ☐ Prefer not to answer
- ☐ Other (please state)

Which of the following areas of your education and career progression do you think the COVID-19 pandemic has or will affect? Please select all that apply

- ☐ Clinical knowledge
- ☐ Anatomy knowledge
- ☐ Systems based knowledge
- ☐ Research opportunities
- ☐ EPM scores
- ☐ Public health knowledge
- ☐ No areas
- ☐ Prefer not to answer
- ☐ Other (please state)

Have you worked or are you currently working within a healthcare setting during the course of this pandemic?

- ○ Yes
- ○ No

Which of the following roles have you taken up whilst working in the hospital?

- ☐ Full time doctor
- ☐ Part time doctor
- ☐ Locum doctor
- ☐ Administrative role
- ☐ Nursing role
- ☐ Healthcare Assistant
- ☐ Receptionist
- ☐ Clinical technician
- ☐ Porter
- ☐ Prefer not to answer
- ☐ Other (please state)

What are the reasons for this?

- ☐ Not enough opportunities
- ☐ Risk of getting infection
- ☐ Risk of passing infection to family members
- ☐ Not interested in getting a job yet
- ☐ Caregiver role
- ☐ Childcare responsibility
- ☐ Underlying medical condition
- ☐ Prefer not to answer
- ☐ Other (please state)

How likely are you to continue in a career in medicine?

- ○ Extremely likely
- ○ Somewhat likely
- ○ Neither likely nor unlikely
- ○ Somewhat unlikely
- ○ Extremely unlikely

How likely are you to practice medicine in the UK?

- ○ Extremely likely
- ○ Somewhat likely
- ○ Neither likely nor unlikely
- ○ Somewhat unlikely
- ○ Extremely unlikely

Have the events of the COVID-19 pandemic influenced your decision to pursue a certain specialty?

- ○ Yes

○ No

How so?

Thank you for completing the survey.

Survey Powered By Qualtrics
