# Supplementary material for: SPICE-19: a 3-Month Prospective Cohort Study of 640 Medical Students and Foundation Doctors
Source: Med Sci Educ. 2021 Jul 21;31(5):1621–37. doi: 10.1007/s40670-021-01349-0 (PMC8294310; doi:10.1007/s40670-021-01349-0)
Supplement: Supplementary file 3 — Supplementary file1 (DOCX 128 kb) [file 40670_2021_1349_MOESM3_ESM.docx]

***Appendix S2:* a list of medical schools recognised by the GMC and listed by the MSC as of 1^st^ April 2020**

The University of Aberdeen

The University of Birmingham

The University of Bristol

The University of Buckingham

The University of Cambridge

Cardiff University

The University of Dundee

The University of East Anglia

The University of Edinburgh

University of Exeter Medical School

The University of Glasgow

The Imperial College of Science, Technology and Medicine

Keele University

King’s College London

Lancaster University

The University of Leeds

The University of Leicester

The University of Liverpool

The University of Manchester

The University of Newcastle

The University of Nottingham

The University of Oxford

Plymouth University Peninsula Schools of Medicine and Dentistry

Queen Mary University of London

The Queen’s University of Belfast

St George's Hospital Medical School

The University of St Andrews

Swansea University

The University of Sheffield

The University of Southampton

University College London

The University of Warwick

A combination of the University of Brighton and the University of Sussex

A combination of the University of Hull and the University of York
